# Type XX Collagen Is Elevated in Circulation of Patients with Solid Tumors

**DOI:** 10.3390/ijms23084144

**Published:** 2022-04-08

**Authors:** Jeppe Thorlacius-Ussing, Christina Jensen, Emilie A. Madsen, Neel I. Nissen, Tina Manon-Jensen, Inna M. Chen, Julia S. Johansen, Hadi M. H. Diab, Lars N. Jørgensen, Morten A. Karsdal, Nicholas Willumsen

**Affiliations:** 1 Biomarkers and Research, Nordic Bioscience A/S, 2730 Herlev, Denmark; chj@nordicbio.com (C.J.); eam@nordicbio.com (E.A.M.); nin@nordicbio.com (N.I.N.); tmj@nordicbio.com (T.M.-J.); mk@nordicbio.com (M.A.K.); nwi@nordicbio.com (N.W.); 2 Department of Biomedical Sciences, University of Copenhagen, 2200 Copenhagen, Denmark; 3 Biotech Research & Innovation Centre (BRIC), University of Copenhagen (UCPH), 2200 Copenhagen, Denmark; 4 Department of Oncology, Copenhagen University Hospital—Herlev and Gentofte, 2730 Herlev, Denmark; inna.chen@regionh.dk (I.M.C.); julia.sidenius.johansen@regionh.dk (J.S.J.); 5 Department of Medicine, Copenhagen University Hospital—Herlev and Gentofte, 2730 Herlev, Denmark; 6 Department of Clinical Medicine, Faculty of Health and Medical Sciences, University of Copenhagen, 2200 Copenhagen, Denmark; larsnjorgensen@hotmail.com; 7 Digestive Disease Center, Bispebjerg Hospital, University of Copenhagen, 2400 Copenhagen, Denmark; hadi.mahmoud.haider.diab@regionh.dk

**Keywords:** cancer, ECM, type XX collagen, serum, biomarker, PDAC

## Abstract

In the tumor microenvironment, the extracellular matrix (ECM) has been recognized as an important part of cancer development. The dominant ECM proteins are the 28 types of collagens, each with a unique function in tissue architecture. Type XX collagen, however, is poorly characterized, and little is known about its involvement in cancer. We developed an ELISA quantifying type XX collagen, named PRO-C20, using a monoclonal antibody raised against the C-terminus. PRO-C20 and PRO-C1, an ELISA targeting the N-terminal pro-peptide of type I collagen, was measured in sera of 219 patients with various solid cancer types and compared to sera levels of 33 healthy controls. PRO-C20 was subsequently measured in a separate cohort comprising 36 patients with pancreatic ductal adenocarcinoma (PDAC) and compared to 20 healthy controls and 11 patients with chronic pancreatitis. PRO-C20 was significantly elevated in all cancers tested: bladder, breast, colorectal, head and neck, kidney, lung, melanoma, ovarian, pancreatic, prostate, and stomach cancer (*p* < 0.01–*p* < 0.0001). PRO-C1 was only elevated in patients with ovarian cancer. PRO-C20 could discriminate between patients and healthy controls with AUROC values ranging from 0.76 to 0.92. Elevated levels were confirmed in a separate cohort of patients with PDAC (*p* < 0.0001). High PRO-C20 levels (above 2.57 nM) were predictive of poor survival after adjusting for the presence of metastasis, age, and sex (HR: 4.25, 95% CI: 1.52–11.9, *p*-value: 0.006). Circulating type XX collagen is elevated in sera of patients with various types of cancer and has prognostic value in PDAC. If validated, PRO-C20 may be a novel biomarker for patients with solid tumors and can help understand the ECM biology of cancer.

## 1. Introduction

Cancer is a major global health problem, and the burden of cancer incidence and mortality is rising worldwide [1]. One part of the problem is the lack of relevant biomarker tools that can, amongst other things, detect cancer earlier and predict survival and response to treatment. Additionally, traditional biomarkers usually involve invasive procedures such as tissue biopsies. A promising non-invasive alternative is liquid biopsies, where biomarkers are measured in the blood and can be assessed easily with minimal discomfort and complications [2]. Examples include measuring the presence of tumor components such as tumor DNA or tumor cells directly [3]. This approach, however, is blind to the influence of the tumor microenvironment, which in recent years has proven to be intricately involved in many important aspects of cancer [4] and components of the tumor microenvironment can be measured in blood [5]. Therefore, a novel and fruitful approach could be to supplement an assessment of the tumor with an assessment of its environment.

The extracellular matrix (ECM) of the tumor microenvironment, defined as the non-cellular part of tissues, has been recognized as an important part of cancer development [6]. When assessing the ECM, one cannot overlook the collagens, because they are collectively some of the most abundant proteins in the human body and have also been implicated in a variety of different processes relevant to cancer: tumor stiffness, tumor immunity, and metastasis [7]. In cancer, the dynamic balance of collagen formation and degradation is knocked askew, and collagen can accumulate to form tumor fibrosis. The collagen superfamily spans 28 different types and most of the collagen cancer research has focused on the abundant and well-characterized collagens, such as type I, III, or IV collagens. As an example, type I collagen is ubiquitous in the tumor microenvironment and is the primary component of tumor fibrosis [8]. In addition to its structural role, type I collagen can activate signaling that promotes invasion [9,10]. Recent reports, however, suggest that collagens, such as type I collagen, can have both pro- and anti-tumor effects [11,12,13].

We have previously shown that these collagens can be used as cancer biomarkers. For example, non-invasive quantification of type III collagen deposition reflects fibrotic activity and is prognostic in several cancers [14]. Seeing this, we pondered the biomarker potential of the other members of the collagen family. Additionally, in contrast to the abundant collagens, very little is known about the role in cancer of the minor and poorly characterized collagens. Nonetheless, a connection between cancer and the minor collagens can be hypothesized because the expression of many minor collagens is exclusive to tissue development, which shares with cancer the activation of signaling pathways and regulation of ECM components [15,16,17]. This is true for the membrane-bound and multiplexin collagens, which have both structural and signaling functions shared between tissue development and cancer, and their expression is correlated with survival in patients with cancer [18]. It appears that these specialized collagens are used for specific purposes, namely the development of tissues and cancer—both involving dramatic tissue remodeling [17]. In support of this hypothesis, we recently discovered elevated levels of a minor collagen, type XIX collagen, in the blood of patients with cancer [19]. However, many minor collagens remain largely unexplored in the cancer context and this prompted the present study.

Type XX collagen is one such unexplored collagen. Based on its structural features, type XX collagen is part of the fibril-associated collagens with interrupted triple helices (FACIT) family. These collagens are thought to associate with the fibrillar collagens to regulate their organization and interactions [20]. Structural features of type XX collagen include several fibronectin type III repeats, a von Willebrand factor A domain, a thrombospondin-like domain, as well as collagenous triple-helix domains, interspersed with non-collagenous domains [20]. Within the FACIT family, type XII and XIV are the closest relatives to type XX collagen [20]. Little is known about the expression, localization, and function of type XX collagen. It was originally cloned from chick embryos in which expression was primarily in corneal epithelium, but detectable in embryonic skin, lung, sternal cartilage, and tendon [20]. RNA expression data from the human protein atlas (https://www.proteinatlas.org/ENSG00000101203-COL20A1/tissue, accessed on 25 February 2022) suggest an enrichment of COL20A1 RNA expression in human brain and minor upticks in testes and spleen tissues [21]. In the context of cancer, previous studies have mostly found type XX collagen in brain cancers. Based on cDNA microarrays, COL20A1 was elevated in so-called brain-tumor initiating cells versus regular glioma cell lines and normal brain astrocytes [22]. In a separate study, using biopsy-derived glioma cell models, a downregulation of COL20A1 RNA was observed after treatment with histone deacetylase inhibitors [23]. Reports on type XX collagen in other cancers are rare, but COL20A1 was included in a 16-gene signature associated with breast cancer recurrence, metastasis, and poor survival in a Chinese population. However, the weight that COL20A1 was assigned in the reported model was minor [24]. Lastly, COL20A1 gene expression is also upregulated in early stage prostate tumorigenesis [25]. These studies interrogate type XX collagen at the RNA or DNA levels, so studies at the protein level and its relevance to cancer are lacking.

In this study, we set out to develop a robust ELISA assay targeting the C-terminus of type XX collagen and use it to quantify levels in serum samples from patients with cancer to evaluate its biomarker potential. This study is the first to report on an assay quantifying type XX collagen and the first to show that, not only can it be measured it blood, but it is elevated in sera of patients with cancer and is prognostic for survival.

## 2. Results

### 2.1. PRO-C20 ELISA Development and Validation

**Figure 1:**
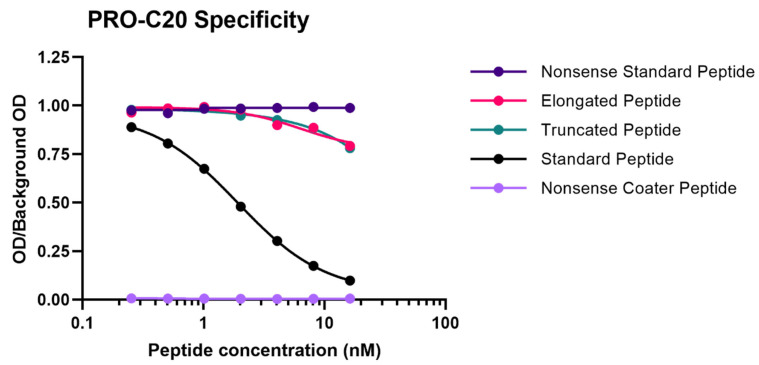
Specificity of the PRO-C20 assay. Inhibition curve of the standard peptide (RHLEGRGEPGAVGQMGSPGQQGASTQGLWE), elongated peptide (QGASTQGLWES), truncated peptide (QGASTQGLW), and a non-sense standard peptide (SHAHQRTGGN) as well as a non-sense coater peptide (Biotin-SHAHQRTGGN). Peptides were diluted twofold in series from 16 nM to 0.25 nM. Signal is shown as the ratio between measured optical density of the sample (OD) and the measured optical density of a blank buffer sample (background OD) as a function of peptide concentration in nM on a logarithmic scale. Points correspond to means of duplicate measurements. Lines correspond to four-parametric logistic regression of each dilution series. Inhibition of the signal indicates competition for antibody binding.

Optimizations of the ELISA protocol included the best time and temperature of incubation, choice of assay buffer, and concentrations of kit components. The settings were chosen based on which gave the best sensitivity in human serum samples whilst upholding the technical requirements outlined below. The chosen format for the ELISA was competitive, so the specificity of the assay was evaluated by the ability of different peptides to compete for binding to the monoclonal antibody. The 10 and 30 amino acid versions of the standard peptide had the exact same behavior (data not shown), so the 30 amino acid version was chosen for the final assay protocol because it is a more stable reagent. Further, the standard peptide dose-dependently inhibited the signal (IC50 = 1.82 nM), whereas the elongated and truncated did not (IC50 = not estimable) (Figure 1). The non-sense coater peptide resulted in no detectable signal as expected. This setup confirms that the monoclonal antibody is specific towards the type XX collagen C-terminus and does not bind closely related peptides.

Other aspects of the technical validation are summarized in Table 1. Accuracy testing using spiking recovery tests revealed excellent recovery of the standard peptide in human sera with an average recovery of 101%. The same was true with matrix-in-matrix spiking where a spiking of a human serum sample into another separate human serum sample resulted in an average recovery of 95%. Interference from the commonly interfering substances hemoglobin, lipids, and biotin was not observed, with recoveries within 15% even for the highest concentrations. Assay variation was below 6% for both inter- and intra-assay variation, thus well below the target values of 10% and 15%, respectively. Analyte stability was evaluated for up to 48 h at either 4 or 20 °C and recoveries were within 15%. Stability following four freeze–thaw cycles was also good, with a recovery within 15%. Linearity of dilution was accepted from undiluted down to 1:2 dilution. At 1:4 dilution, the recovery of human serum samples dropped below the acceptance limit of 80% analyte recovery. Together these results indicate that PRO-C20 is an accurate, precise, and robust assay.

### 2.2. PRO-C20 in Serum of Patients with Solid Cancers

**Figure 2:**
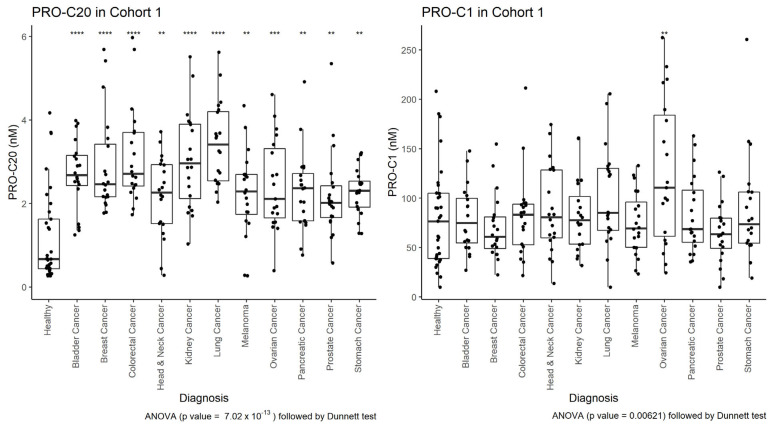
PRO-C20 (**left**) and PRO-C1 (**right**) in cohort 1. Quantification of PRO-C20 and PRO-C1 in serum from healthy controls (*n* = 33), bladder cancer (*n* = 20), breast cancer (*n* = 20), colorectal cancer (*n* = 20), head and neck cancer (*n* = 20), kidney cancer (*n* = 20), lung cancer (*n* = 20), melanoma (*n* = 20), ovarian cancer (*n* = 19), pancreatic cancer (*n* = 20), prostate cancer (*n* = 20), and stomach cancer (*n* = 20). Biomarker levels are presented as Tukey-style boxplots with data-point jitter. Samples measuring below the lower limit of measurement range were given the value of that limit, as determined in the validation of the assays. Differences in biomarker levels between cancer groups and the healthy controls were evaluated by ordinary ANOVA followed by multiple comparisons to the controls with Dunnett test. **** indicates a *p*-value below 0.0001. *** below 0.001. ** below 0.01.

**Figure 3:**
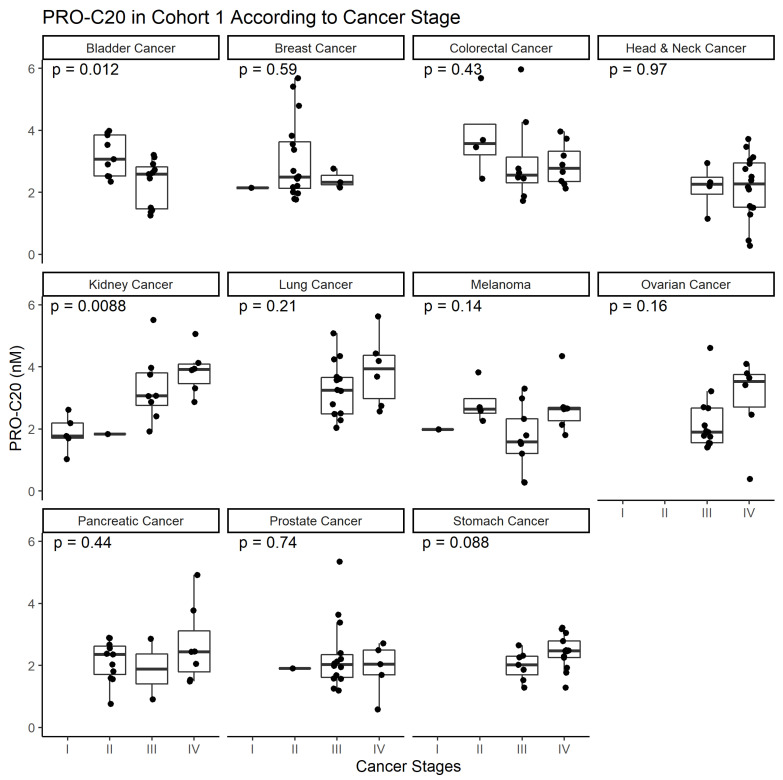
PRO-C20 in cohort 1 according to cancer stage. Quantification of PRO-C20 in cohort 1 was stratified into cancer types and plotted as a function of cancer stage. Biomarker levels are presented as Tukey-style boxplots with datapoint jitter. Differences in PRO-C20 according to cancer stage in each cancer type was evaluated by ordinary ANOVA.

To evaluate the biological relevance of circulating type XX collagen, we measured PRO-C20 in a cohort of patients with solid cancers diagnosed with 11 different solid tumors. As a comparison to a well-described assay of an abundant protein, we also measured PRO-C1 in the same samples. Serum levels of the markers in these patients were compared to serum levels in healthy controls. The cohort characteristics are summarized in Table 2. For PRO-C20, nearly all samples measured within the measurement range, indicating that the settings found during assay development and the limits set during validation were applicable for quantifying these samples. Further, for all cancers tested, there was a substantial dispersion in PRO-C20 levels within each group, indicating that the assay has an appropriate dynamic range for quantifying serum samples from cancer patients. PRO-C20 levels were significantly elevated in all cancers tested compared to the healthy controls (Figure 2). The median PRO-C20 levels were three to five-fold higher in serum collected from patients with cancer compared to healthy controls. For PRO-C1, in comparison, only the ovarian cancer group was significantly elevated compared to healthy controls and the largest fold increase in median levels compared to healthy controls was 1.4-fold in ovarian cancer. For some cancer types, PRO-C20 trended upwards with increasing cancer stage (Figure 3). PRO-C1 did not seem to be associated with stage of disease (data not shown). PRO-C20 proved capable of discriminating between healthy and cancer, as evidenced by the AUROC values ranging from 0.76 to 0.92 (Table 3). Overall, these results suggest that circulating levels of PRO-C20 and type XX collagen are elevated in a wide variety of cancer types.

### 2.3. PRO-C20 in Serum of Patients with PDAC

**Figure 4:**
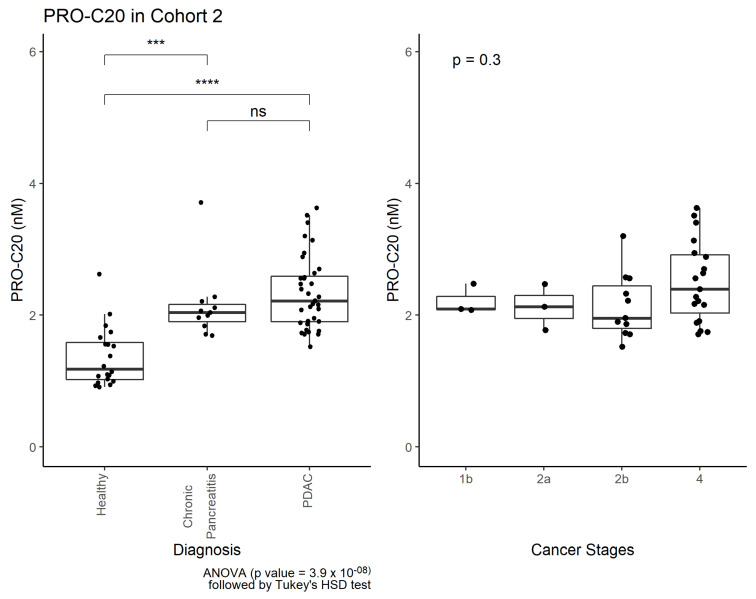
PRO-C20 in cohort 2. **Left**: Quantification of PRO-C20 in sera from healthy controls (*n* = 20), chronic pancreatitis (*n* = 11), and pancreatic ductal adenocarcinoma (PDAC, *n* = 36). PRO-C20 levels are presented as Tukey-style boxplots with data-point jitter. Differences in PRO-C20 levels between the groups were evaluated by ordinary ANOVA followed by multiple comparisons with Tukey’s HSD test. **** indicates a *p*-value below 0.0001. *** below 0.001. ns no significance. **Right**: Quantification of PRO-C20 in cohort 2 plotted as a function of cancer stage. Biomarker levels are presented as Tukey-style boxplots with datapoint jitter. Differences in PRO-C20 according to cancer stage were evaluated by ordinary ANOVA.

PRO-C20 was measured in a cohort of patients with pancreatic ductal adenocarcinoma (PDAC), patients with chronic pancreatitis, and healthy controls. Cohort characteristics are summarized in Table 4. PRO-C20 was significantly elevated in the sera of patients with PDAC and chronic pancreatitis compared to healthy controls (Figure 4). There was no significant difference between PDAC and chronic pancreatitis. PRO-C20 proved capable of discriminating between healthy and diseased samples, as demonstrated by the AUROC values of 0.92 and 0.91 of PDAC and chronic pancreatitis, respectively (Table 5). PRO-C20 could only discriminate between PDAC and chronic pancreatitis with an AUROC of 0.63. Although stage four of PDAC had higher median levels there was no clear association between PRO-C20 and cancer stages (Figure 4, right) as well as no association with performance score or different sites of metastasis (data not shown).

**Figure 5:**
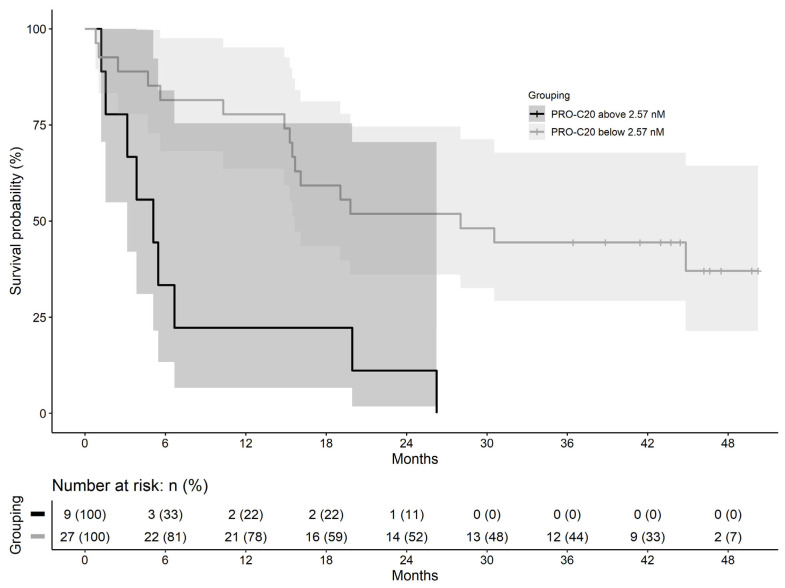
Kaplan–Meier curves of survival in patients with pancreatic ductal adenocarcinoma grouped into high (above 2.57 nM) or low PRO-C20 (below 2.57 nM) shaded with 95% confidence intervals.

A cut-off of 2.57 nM was identified to stratify the PDAC patients into PRO-C20 high and low groups, assigning 25% of patients to the ‘PRO-C20 high’-group. The ‘PRO-C20 low’-group had a median survival time of 853 days, whereas the ‘PRO-C20 high’-group had a median survival time of 155 days (Figure 5). After adjusting for metastasis, age, and sex, high PRO-C20 levels were independently associated with poor overall survival with a hazard ratio of 4.25 (95%CI: 1.52 to 11.9, *p*-value: 0.006, Table 6). When PRO-C20 was instead included in the model as a continuous variable, it was a borderline significant predictor of survival (data not shown). In summary, these results indicate that high PRO-C20 levels are associated with poor overall survival independently of patient demographics and stage of disease.

## 3. Discussion

To study type XX collagen and its role in cancer, we developed the PRO-C20 ELISA. The assay proved to be a sensitive assay, capable of detecting nanomolar amounts of type XX collagen. It was also specific, as evidenced by the epitope specificity of the antibody, and it was accurate in the complex sample matrix of serum. Lastly, the analyte and reagent stability of the assay indicate that it is a robust assay. PRO-C20 was elevated in all cancers tested and although a downward trend in bladder and upward trend in kidney cancer was seen as a function of cancer stage, overall PRO-C20 levels did not seem to be associated with cancer stage. Although not the aim of this study, PRO-C20 proved excellent at discriminating between sera from healthy controls and patients with cancer with large AUC values, but was limited in terms of its diagnostic specificity, seeing as a substantial proportion of the healthy individuals would be misclassified using the cutoffs described in the current study. Future studies should pinpoint the discriminatory performance of PRO-C20 and do so with more clinically relevant control subjects, e.g., individuals with comorbidities such as other chronic diseases.

Compared to PRO-C20, PRO-C1 was not effective at discriminating between sera from healthy controls and patients with cancer. Although type I collagen is elevated in several cancers compared to healthy controls [26], the epitope that the PRO-C1 assay targets is mostly associated with bone metastasis [27] which the patients included here do not have. Part of the reason to include PRO-C1 in the analysis here was to make a comparison to a well-described and abundant protein. This comparison highlights the merit of investigating the biomarker potential of the otherwise poorly characterized collagens of low abundance. This study also lends merit to the hypothesis that the minor collagens are deregulated in disease and may be more pathology-relevant than abundant proteins. Collagens in general can be predictive of response to treatment [13] and we have previously demonstrated how non-invasive quantification of fibrillar collagens, such as type III collagen, can be used to assess fibrotic activity from the periphery and is prognostic in several cancers [14] and predictive of immunotherapy response [5]. The current study builds upon these discoveries and expands the potential of the approach by demonstrating that the breadth of the collagen family may also have biomarker potential.

Interestingly, PRO-C20 was also elevated in patients with chronic pancreatitis, indicating PRO-C20 is released to circulation as a function of inflammation and fibrosis. Chronic pancreatitis is a well-known risk factor of cancer. In fact, it can develop as a result of an underlying, undiagnosed, pancreatic cancer [28], and chronic pancreatitis is characterized by fibrosis and inflammation, similar to cancer, making it difficult to distinguish between the two pathologies [28]. Based on our data, PRO-C20 could not separate the two pathologies, suggesting PRO-C20 is not only released to circulation as a result of cancer, but as a result of more general tissue remodeling. Instead, a context of use for PRO-C20 could be as a PDAC prognostic biomarker, where high PRO-C20 levels were associated with poor overall survival. PDAC has a high mortality rate and a prognostic biomarker in this context could, for example, identify the patients where a comprehensive and potentially toxic therapy regimen may not be appropriate. Follow-up studies should be performed to confirm the findings and further delineate the context of use for PRO-C20.

The function of FACITs such as type XX collagen is unclear. Much of the understanding of how FACITs function is derived from biochemical experiments of type IX and XII collagens. These FACITs bind to the collagen fibrils with their C-terminal region, whereas the N-terminal region projects out from the fibril to interact with other components of the ECM [29,30]. In this way, FACITs can mediate within and between fibril interactions and be seen as organizers of the collagenous ECM and therefore intricately involved in establishing matrix and tissue structure. Type XX collagen shares many structural features with type XII collagen, including the domain structure of both its C-terminal and N-terminal regions, so it is likely that they share a similar function of integrating collagen fibrils. The connection of type XX collagen to fibrillar collagens is not insignificant: changes in the ECM and collagen composition as a result of cancer has been shown to influence tumor progression, metastasis, and clinical outcome of patients [7]. Additionally, the minor collagens associated with the fibrillar ones are important for the formation of new fibrils [31]. Formation of new and remodeling of existing fibrils is relevant to the formation of so-called pre-metastatic niches, wherein the ECM of a metastatic site is remodeled to facilitate the metastasizing cancer cells [32]. Although we did not see a clear association with PRO-C20 levels and cancer stages, several other members of the FACIT family of collagens seem to be regulated as part of the metastatic cascade: type XII collagen is found upregulated at the invasive front of colon cancer cells [33] and type XIX collagen is degraded prior to the degradation of the basement membrane and intravasation [34].

**Figure 6:**
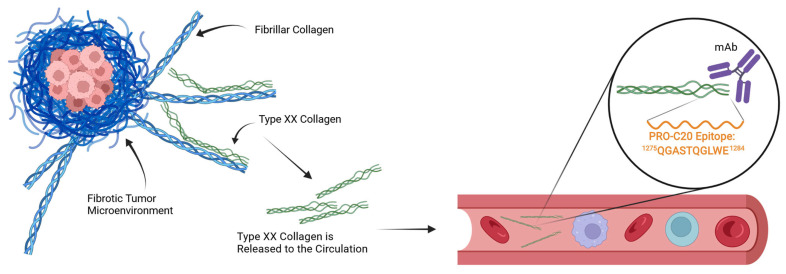
The tumor microenvironment accumulates collagens and forms fibrosis. The primary components of tumor fibrosis are fibrillar collagens, such as type I or type III collagen, and FACIT collagens, such as type XX collagen, are thought to organize the fibrillar collagens. Fragments of type XX collagen find their way to the circulation, where they can be detected in blood samples by a monoclonal antibody (mAb) raised against the C-terminus of type XX collagen and quantified in an ELISA. Figure made with BioRender.

In the case of PRO-C20, which targets the natural C-terminus of the protein, the mechanism of its release to circulation is unclear. Intuitively, a full-length and intact type XX collagen protein would be expected to remain in the tissue to exert its role in integrating collagen fibers. Conversely, a fragmented protein would lose one or more points of interaction with other proteins and is thus less likely to be retained and more likely to be found in circulation (Figure 6). It is therefore possible that PRO-C20 measures fragments and reflects the degradation of type XX collagen, and may explain why the conclusions drawn from our quantification of type XX collagen differs from some of the gene expression studies outlined in the introduction. This difference could be important, because fragmentation of type XX collagen could be a way to downregulate the integration of fibrillar collagens and possibly expose them to further degradation and remodeling. If true, the fragmentation of type XX collagen and the release of the PRO-C20 epitope could reflect remodeling of the fibrillar collagens. The mechanics of this process are not well described, and the quantification approach presented in this study is therefore limited in this respect. Further studies into the processing and release of type XX collagen are needed.

Research on the role of type XX collagen in cancer is very limited, but looking to its closest collagen relative, type XII collagen, reveals some interesting perspectives for the further study of type XX collagen. Studies of skin and cornea reveal that FACITs can regulate collagen fibril diameter and density [35,36]. More and thicker fibrils are associated with an overall stiff matrix, which is a well-known characteristic of cancer [17]. A stiff matrix can, amongst other things, prevent the host immune cells from attacking the tumor cells [37]. Further, type XII collagen is secreted by cancer-associated fibroblasts and colon cancer cells at the invasive front of lesions and is associated with myofibroblastic differentiation [33,38] and involved in TGFβ signaling [35,39]. It is intriguing how our current study supports an important role for another FACIT collagen in the context of cancer, even if the current study provides no clear insight into the mechanisms of this role.

Recently, collagen binding proteins have been used in conjunction with immune checkpoint inhibitors in the form of fusion constructs to target the therapy towards the tumor and limit adverse effects. For example, the collagen-binding domain of von Willebrand Factor fused to checkpoint inhibitor antibodies can concentrate therapy effects towards the fibrotic stroma of the tumor microenvironment [40]. Minor collagens, which bind fibrillar collagens may be useful in a similar way. The interaction partners of type XX collagen are not currently known, but if they are similar to type XII collagen, it is possible that they interact with major fibrillar collagens such as type I collagen and may, therefore, be useful as a homing device to deliver therapies to fibrotic tumors.

The limitations of this study are substantial. Sample sizes for both cohorts are small, increasing the likelihood of bias and false positives. Thankfully, effect sizes seemed large, with large fold-increases in PRO-C20 levels in samples from cancer patients compared to healthy controls. Prognostic evaluation of PRO-C20 was also limited by the small sample size and the dichotomization of biomarker levels, which can introduce bias [41]. Clinical information was especially lacking for the first cohort, limiting the analysis to a basic comparison between indications with little consideration for their clinical outcome. This underlines the need to validate the current findings in a secondary and well-characterized cohort. The goal of this study was to demonstrate the biological relevance and biomarker potential of type XX collagen and a robust assay to quantify it in complex samples. This study was merely one of our first steps to explore the interesting biology of FACIT collagens. The next step for the further validation of PRO-C20 involves confirming the findings presented here in another cohort of sufficient size to determine the cutoffs needed in both a diagnostic and prognostic settings. Special attention should be paid to the comparison to controls, which could preferably be individuals with other chronic diseases than cancer to evaluate, e.g., Chronic pancreatitis to PDAC—which is a comparison more applicable to the clinic. Further, how PRO-C20 levels are affected by treatment or other clinically relevant factors should be evaluated. It seems clear, however, that PRO-C20 is a biomarker with the potential to make an impact for patients in the future, and further investigation into PRO-C20 and the biology of the minor collagens is warranted.

## 4. Materials and Methods

### 4.1. Generation of Monoclonal Antibodies Targeting the PRO-C20 Epitope

A 10 amino-acid peptide ^1275^QGASTQGLWE^1284^ corresponding to the C-terminus of type XX collagen (UniprotKB: Q9P218) was purchased from Genscript (Piscataway, NJ, USA) and used for immunization. This sequence was incorporated into an immunogenic peptide (KLH-CGG-QGASTQGLWE) by covalently cross-linking the target peptide to Keyhole Limpet Hemocyanin (KLH) carrier protein using sulfosuccinimidyl 4-(N-maleimidomethyl) cyclohexane-1-carboxylate (SMCC, Thermo Scientific, Waltham, MA, USA, cat. no. 22322). Glycine and cysteine residues were added at the N-terminal end of the peptide to ensure correct linking of the carrier protein. Monoclonal antibodies were generated by subcutaneous immunization of six-week-old Balb/C mice with 200 µL emulsified antigen containing 100 µg immunogenic peptide mixed with Sigma Adjuvant System (Sigma cat. no. S6322). Consecutive immunizations were performed at 2-week intervals until stable sera titer levels were reached. The mouse with the highest titer was rested for four weeks and was then boosted with 100 µg immunogenic peptide in 100 µL 0.9% NaCl solution intravenously. Hybridoma cells were produced by fusing spleen cells with SP2/0 myeloma cells. The resultant hybridoma cells were then cultured in 96-well microtiter plates and limited dilution was used to secure monoclonal growth. The best antibody clone for the epitope of interest was selected based on a preliminary competitive ELISA for the reactivity towards the selection peptide (the target peptide, QGASTQGLWE), and not an elongated peptide (QGASTQGLWES), a truncated peptide (QGASTQGLW), or a non-sense KLH-conjugated peptide (IRQCPDRTYG-GGC-KLH). The monoclonal antibodies were purified using protein-G-columns according to the manufacturer’s instructions (GE Healthcare Life Sciences, Little Chalfont, UK, cat. no. 17-0404-01). The purified antibodies were labeled with horseradish peroxidase (HRP) using a peroxidase labeling kit (Roche Diagnostics GmbH, Mannheim, Germany, cat. no. 11829696001).

### 4.2. PRO-C20 ELISA Protocol

Several optimizations were made to the ELISA, including the choice of assay buffer, incubation time and temperature, as well as concentrations of antibody and peptides. The final PRO-C20 protocol was performed as follows: a 96-well streptavidin-coated ELISA plate was coated with 100 µL/well of 1.25 ng/mL biotinylated QGASTQGLWE peptide dissolved in assay buffer (25 mM tris-buffered saline (TBS), 1% bovine serum albumin (BSA) (*w*/*v*), 0.1% Tween-20 (*w*/*v*), 2 g/L NaCl, pH 8.0) and incubated for 30 min at 20 °C with shaking at 300 RPM. After washing five times with washing buffer (25 mM Tris, 50 mM NaCl, pH 7.2), 20 µL/well of sample was added in duplicates followed by 100 µL/well of 50 ng/mL HRP-labelled monoclonal antibody in assay buffer and incubated for 1 h at 20 °C with shaking at 300 RPM. After a second washing cycle, 100 µL/well of 3, 3′, 5, 5′-tetramethylbenzidine (TMB) was added and incubated for 15 min in darkness at 20 °C with shaking at 300 RPM. The reaction was stopped by adding 100 µL/well of 1% H_2_SO_4_ (*v*/*v*). Absorbance was measured at 450 nm with 650 nm as reference. To generate a standard curve, 20 µL/well of 50 ng/mL RHLEGRGEPGAVGQMGSPGQQGASTQGLWE peptide, serially diluted twofold, was added to appropriate wells and a four-parametric logistic regression model was used to fit a curve. Each plate included five quality control samples comprising one human serum, one horse serum, one human plasma, and two standard peptide in assay buffer samples to monitor intra- and inter-assay variation.

### 4.3. Technical Validation of the PRO-C20 ELISA

Antibody specificity was evaluated by the inhibition of signal by two different versions of the standard peptide: a 10 amino acid version (QGASTQGLWE) and 30 amino acid version (RHLEGRGEPGAVGQMGSPGQQGASTQGLWE), both tested in twofold dilution series. In addition, an elongated (QGASTQGLWES) and truncated peptide (QGASTQGLW) of the PRO-C20 epitope was tested, as well as a non-sense standard peptide (SHAHQRTGGN) and a non-sense coater peptide (Biotin-SHAHQRTGGN) with sequences corresponding to the C-terminus of type XIX collagen. Linearity of dilution was evaluated by twofold dilutions of human serum samples and then calculating the percentage recovery of the measured concentration relative to the predicted concentration. Accuracy was evaluated by either spiking a known quantity of the standard peptide into human serum samples or by spiking one human serum sample into another human serum sample at different ratios (100:0, 75:25, 50:50, 25:75, or 0:100), followed in both cases by calculating the percentage recovery of the spiked sample relative to the non-spiked sample. The influence of commonly interfering substances including hemoglobin, lipids, and biotin were evaluated by spiking human serum samples with a known quantity of the interfering substances (hemoglobin low = 2.5 mg/mL, high = 5 mg/mL; lipids low = 1.5 mg/mL, high = 5 mg/mL; and biotin low = 5 ng/mL, high = 100 ng/mL) and calculating the percentage recovery relative to the non-spiked sample. Assay variation was tested by running ten independent runs of the assay using ten quality control samples in double determinations. Five quality control samples were one human serum, one horse serum, one human plasma, and five samples of standard peptide in assay buffer of varying concentrations. Intra-assay variation was calculated as the mean coefficient of variance (CV%) between double determinations during each run of the assay. Inter-assay variation was calculated as the mean CV% between all determinations across all ten runs. Lower- and upper-limit of the measurement range were determined as the concentrations that denote the limits of the linear range of the assay, defined as the intersection between the line drawn through the standard points that comprise the linear range of the assay and the line drawn horizontally at either the maximum or minimum OD. Lower limit of detection was calculated as the mean interpolated concentration of 21 blank samples only containing assay buffer plus three standard deviations. Upper limit of detection was calculated as the mean interpolated concentration of standard peptide corresponding to the highest concentration of the standard curve minus three standard deviations. Analyte stability was evaluated for three human serum samples incubated at either 4 or 20 °C for 2, 4, 24, or 48 h and calculating the percentage recovery of the incubated samples relative to the corresponding control sample kept at −20 °C. Freeze–thaw stability was evaluated by repeatedly freezing and thawing human serum samples for up to 4 cycles and calculating the percentage recovery of the cycled samples relative to the corresponding control samples that underwent a single freeze–thaw cycle.

### 4.4. PRO-C1 ELISA Protocol

The PRO-C1 ELISA (Nordic Bioscience, Herlev, Denmark, cat. no. 2800) measures the N-terminal pro-peptide of type I collagen, sometimes called PINP in the literature. PRO-C1 levels reflect type I collagen formation, as the release of the pro-peptide is a critical step in the maturation process of type I collagen. A detailed description and protocol of the ELISA has already been published [42].

### 4.5. Patient Samples

The first cohort included serum samples from 219 patients with cancer and 33 healthy controls. It included 10 groups, each with 20 patients with bladder, breast, colorectal, head and neck, kidney, lung, pancreatic, prostate, and stomach cancer or melanoma. In addition, 19 patients with ovarian cancer and 33 age-matched healthy controls were included. Serum samples from cancer patients and healthy controls were obtained from Proteogenex (Los Angeles, CA, USA) and BioIVT (Westbury, NY, USA), respectively. Samples were stored at −80 °C prior to analysis. A summary of the cohort characteristics can be found in Table 2. According to the vendors, sample collection was approved by an Institutional Review Board or Independent Ethical Committee and patients gave their informed consent: Russian Oncological Research Centre n.a. Blokhin RAMS (PG-ONC 2003/1) and Western Institutional Review Board, Inc. (WIRB^®^Protocol #20161665). All investigations were carried out according to the Helsinki Declaration.

The second cohort included serum samples from 36 patients with PDAC, 11 patients with chronic pancreatitis, and 20 healthy controls. Serum samples from healthy controls were obtained from Valley Biomedical (Winchester, VA, USA). Samples were stored at −80 °C prior to analysis. All patients with PDAC and chronic pancreatitis were included in the Danish BIOPAC study (ClinicalTrials.gov ID: NCT03311776) and were collected prospectively prior to treatment. A detailed description of the cohort has already been published elsewhere [43]. A summary of the cohort characteristics can be found in Table 4.

### 4.6. Statistics

Comparisons of biomarker levels across groups were investigated using ordinary one-way ANOVA. In cohort 1, ANOVA was followed by pair-wise comparisons to the control group using the Dunnett test. In cohort 2, ANOVA was followed by Tukey’s Honestly Significant Difference Test. Differences in PRO-C20 levels across cancer stages were evaluated by ordinary one-way ANOVA. Diagnostic accuracy was tested by ROC curve analysis, including the area under the curve as well as sensitivity, specificity, positive predictive value, and negative predictive value at the PRO-C20 cutoff where the Youden-index was maximized. In the survival analysis, a cut-off value was defined using the maximally selected rank statistic and used to stratify patients into PRO-C20 high and low groups. Survival of these two groups was estimated using Kaplan–Meier curves. Cox proportional hazards regression was used to model survival and estimate the hazard ratios. A *p*-value below 0.05 was considered statistically significant. Asterisks indicate the following significance levels: * *p* < 0.05; ** *p* < 0.01; *** *p* < 0.001; and **** *p* < 0.0001. Statistical analysis and graphs were compiled in GraphPad Prism (version 9.1.0 for Windows, GraphPad Software, San Diego, CA, USA, www.graphpad.com, accessed on 17 March 2022) and R version 4.0.4 (R Core Team (2021), R Foundation for Statistical Computing, Vienna, Austria, https://www.R-project.org, accessed on 17 March 2022).

## 5. Conclusions

In this study, an ELISA to quantify the presence of type XX collagen in blood was successfully developed, optimized, and validated. PRO-C20 was technically robust, accurate, and sensitive. The levels of circulating type XX collagen could be assessed in the sera of patients with cancer and healthy controls, with levels of PRO-C20 being significantly higher in patients with cancer. Subsequent confirmation of these elevated levels was obtained in sera of patients with PDAC and high PRO-C20 levels were associated with poor overall survival. This discovery suggests that PRO-C20 has potential as a biomarker assay and warrants further investigation into the role of type XX collagen in cancer.

## Figures and Tables

**Table 1:** Summary of PRO-C20 validation tests.

Test	Result
IC50	1.82 nM
Measurement range	0.84–27.3 nM
Detection range	0.20–14.9 nM
Dilution recovery of human serum (undiluted to 1:2)	86.8%
Spiking recovery of peptide in serum	101.3%
Spiking recovery of serum in serum	95.5%
Hemoglobin interference recovery, low/high conc.	104.9/103.7%
Lipids interference recovery, low/high conc.	103.2/109.2%
Biotin interference recovery, low/high conc.	98.1/85.4%
Inter-assay variation	5.4%
Intra-assay variation	5.9%
Analyte stability (48 h 4 °C/48 h 20 °C)	89.5/87.1%
Freeze–thaw stability up to four cycles	89.8%

**Table 2:** Clinicopathological characteristics of cohort 1.

Characteristic	Cancer, *n* = 219	Healthy, *n* = 33
**Diagnosis, *n* (%)**		
Bladder Cancer	20 (9.1)	-
Breast Cancer	20 (9.1)	-
Colorectal Cancer	20 (9.1)	-
Head and Neck Cancer	20 (9.1)	-
Kidney Cancer	20 (9.1)	-
Lung Cancer	20 (9.1)	-
Melanoma	20 (9.1)	-
Ovarian Cancer	19 (8.7)	-
Pancreatic Cancer	20 (9.1)	-
Prostate Cancer	20 (9.1)	-
Stomach Cancer	20 (9.1)	-
Healthy	-	33 (100)
**Cancer Stages, *n* (%)**		
I	7 (3.2)	-
II	46 (21)	-
III	93 (42)	-
IV	73 (33)	-
**Age, Mean (SD)**	59 (11)	58 (6)
**Sex, *n* (%)**		
Male	119 (54)	21 (64)
Female	100 (46)	12 (36)

**Table 3:** PRO-C20 in the comparison between cancers and healthy controls in cohort 1. AUC: Area Under the Curve. Cutoff: the cutoff value of PRO-C20 in nM where the Youden Index was maximized. Youden: The Youden Index at the PRO-C20 cutoff. PPV: Positive Predictive Value. NPV: Negative Predictive Value.

Diagnosis	AUC	Cutoff	Youden	Sensitivity	Specificity	PPV	NPV
Lung Cancer	0.92	2.03	0.82	1.00	0.82	0.77	1.00
Colorectal Cancer	0.90	1.73	0.76	1.00	0.76	0.71	1.00
Kidney Cancer	0.88	1.71	0.71	0.95	0.76	0.70	0.96
Breast Cancer	0.87	1.77	0.76	1.00	0.76	0.71	1.00
Bladder Cancer	0.86	2.35	0.65	0.80	0.85	0.76	0.88
Stomach Cancer	0.83	1.28	0.64	1.00	0.64	0.62	1.00
Pancreatic Cancer	0.82	0.77	0.61	1.00	0.61	0.61	1.00
Ovarian Cancer	0.80	1.41	0.61	0.95	0.67	0.62	0.96
Prostate Cancer	0.79	1.19	0.59	0.95	0.64	0.61	0.95
Head and Neck Cancer	0.76	1.15	0.54	0.90	0.64	0.60	0.91
Melanoma	0.76	1.53	0.55	0.85	0.70	0.63	0.88

**Table 4:** Clinicopathological characteristics of cohort 2.

Characteristic	PDAC, *n* = 36	Pancreatitis, *n* = 11	Healthy, *n* = 20
**Age, Mean (SD)**	66 (8)	61 (9)	57 (6)
**Sex, *n* (%)**			
Female	17 (47)	1 (9.1)	10 (50)
Male	19 (53)	10 (91)	10 (50)
**BMI, Mean (SD)**	23.7 (3.7)	-	-
**Diabetes, *n* (%)**	7 (19)	3 (27)	-
**Tobacco, *n* (%)**			
Ever	23 (64)	8 (73)	-
Never	13 (36)	3 (27)	-
**Stage, *n* (%)**			
1b	3 (8.3)	-	-
2a	3 (8.3)	-	-
2b	11 (31)	-	-
4	19 (53)	-	-
**Metastases, *n* (%)**			
Liver Metastasis	15 (79)	-	-
Other Metastasis	4 (21)	-	-
**Performance Status, *n* (%)**			
0	15 (45)	-	-
1	14 (42)	-	-
2	4 (12)	-	-
Unknown	3	-	-

**Table 5:** PRO-C20 in the comparison between PDAC and healthy controls, chronic pancreatitis, and healthy controls or PDAC and chronic pancreatitis in cohort 2. AUC: Area Under the Curve. Cutoff: the cutoff value of PRO-C20 in nM where the Youden Index was maximized. Youden: The Youden Index at the PRO-C20 cutoff. PPV: Positive Predictive Value. NPV: Negative Predictive Value.

Positive Classifier	Negative Classifier	AUC	Cutoff	Youden	Sensitivity	Specificity	PPV	NPV
PDAC	Healthy	0.92	1.71	0.77	0.97	0.80	0.90	0.94
Pancreatitis	Healthy	0.91	1.69	0.80	1.00	0.80	0.73	1.00
PDAC	Pancreatitis	0.63	2.33	0.35	0.44	0.91	0.94	0.33

**Table 6:** Multivariate cox regression of survival using PRO-C20 in combination with clinical characteristics in 36 patients with pancreatic ductal adenocarcinoma.

Characteristic	HR (95% CI) ^1^	*p*-Value
PRO-C20: Above 2.57 nM	4.25 (1.52 to 11.9)	0.006
Metastasis: Yes	5.00 (1.93 to 13.0)	<0.001
Age: Continuous	1.08 (1.01 to 1.15)	0.025
Sex: Male	2.71 (1.13 to 6.48)	0.025

*n* = 36; *n* events = 25; R^2^ = 0.549; c-index = 0.808; c-index SE = 0.036. ^1^ HR = Hazard Ratio, CI = Confidence Interval.

## Data Availability

The data presented in this study are available on request from the corresponding author.

## Abbreviations

ECM (Extracellular Matrix); PDAC (Pancreatic Ductal Adenocarcinoma); FACIT (Fibril-Associated Collagen with Interrupted Triple Helices); Optical Density (OD); Horseradish Peroxidase (HRP); Tris-buffered Saline (TBS); Bovine Serum Albumin (BSA); 3, 3′, 5, 5′-tetramethylbenzidine (TMB).
